# A randomized controlled efficacy trial of an electronic screening and brief intervention for alcohol misuse in adolescents and young adults vulnerable to HIV infection: step up, test up study protocol

**DOI:** 10.1186/s12889-020-8154-6

**Published:** 2020-01-08

**Authors:** Lisa M. Kuhns, Niranjan Karnik, Anna Hotton, Abigail Muldoon, Geri Donenberg, Kristin Keglovitz, Moira McNulty, John Schneider, Faith Summersett-Williams, Robert Garofalo

**Affiliations:** 1 The Potocsnak Family Division of Adolescent and Young Adult Medicine, Ann & Robert H. Lurie Children’s Hospital, 225 E. Chicago Avenue, Box 161, Chicago, IL 60611 USA; 2 Department of Pediatrics, Northwestern University, Feinberg School of Medicine, Chicago, IL USA; 3 Department of Psychiatry & Behavioral Sciences, Rush Medical College, Rush University, Chicago, IL USA; 4 Chicago Center for HIV Elimination, University of Chicago Medicine, Chicago, IL USA; 5 Department of Medicine, University of Illinois at Chicago, Center for Dissemination and Implementation Science, Chicago, IL USA; 6 Howard Brown Health, Chicago, IL USA; 7 Department of Psychiatry, Northwestern University, Feinberg School of Medicine, Chicago, IL USA

**Keywords:** HIV prevention, Men who have sex with men, Transgender women, Alcohol intervention

## Abstract

**Background:**

Young people account for more than a quarter of new HIV infections in the US, with the majority of cases among young men who have sex with men; young transgender women are also vulnerable to infection. Substance use, particularly alcohol misuse, is a driver of sexual transmission and a potential barrier to engagement in the HIV prevention and care continuum, however vulnerable youth are difficult to reach for substance use services due, in part, to complex social and structural factors and limited access to health care. The Community Prevention Services Task Force recommends electronic screening and brief intervention as an evidence-based intervention for the prevention of excessive alcohol consumption; however, no prior studies have extended this model to community-based populations of youth that are susceptible to HIV infection. This paper describes the study protocol for an electronic screening and brief intervention to reduce alcohol misuse among adolescents and young adults vulnerable to HIV infection in community-based settings.

**Methods:**

This study, Step Up, Test Up, is a randomized controlled trial of an electronic alcohol screening and brief intervention among youth, ages 16–25, who are vulnerable to HIV infection. Individuals who present for HIV testing at one of three community-based locations are recruited for study participation. Eligibility includes those aged 16–25 years, HIV-negative or unknown HIV status, male or trans female with a history of sex with men, and English-speaking. Participants who screen at moderate to high risk for alcohol misuse on the Alcohol Use Disorders Identification Test (AUDIT) are randomized (1:1) to either an electronic brief intervention to reduce alcohol misuse or a time-and attention-matched control. The primary outcome is change in the frequency/quantity of recent alcohol use at 1, 3, 6 and 12-month follow-up.

**Discussion:**

Testing of evidence-based interventions to reduce alcohol misuse among youth vulnerable to HIV infection are needed. This study will provide evidence to determine feasibility and efficacy of a brief electronically-delivered intervention to reduce alcohol misuse for this population.

**Trial registration:**

ClinicalTrials.gov number, NCT02703116, registered March 9, 2016.

## Background

Recent estimates of U.S. HIV incidence indicate that young people aged 13 to 24 account for nearly a quarter of new infections, with the majority of cases among young men who have sex with men (YMSM) [1, 2]. While official incidence statistics are not differentiated by transgender status, local estimates indicate extremely high rates of unrecognized HIV infection among young transgender women (YTW) as well [3–5]. Substance use, particularly alcohol misuse, has been identified as a driver of sexual transmission of HIV infection [6–9] and a potential barrier to engagement in HIV prevention and care among YMSM and YTW, however these youth are difficult to reach for substance use services due, in part, to complex social and structural factors and limited access to health care. YMSM and YTW, however, often seek HIV testing and other supportive services in community-based and outreach settings. These settings are underutilized as potential entry points for engagement in comprehensive care across the HIV prevention and care continuum, including pre-exposure prophylaxis (PrEP) for HIV-negative youth who are at risk of HIV acquisition.

Given the barriers to substance use screening and intervention among YMSM and YTW, interventions are needed that are client-centered and can be deployed in community settings. Motivational interviewing (MI) is a well-established client-centered behavioral change approach [10, 11]. The theoretical basis for MI draws in part on Rogerian concepts of psychotherapy that emphasize the need for self-exploration and patient-centeredness. The core of MI emphasizes that individuals must be empowered to change and that given the right set of circumstances they will gravitate toward more pro-social outcomes. The hallmarks of this method avoid telling individuals what to do, and instead create a space where the individual is allowed to explore their motivations through discrepancies that exist in their life (i.e. alcohol makes me feel relaxed, but I don’t like the way I treat others when I drink). By seeing these discrepancies, individual find a motivation to change and then move forward.

Expansion of MI-based or MI-informed approaches is taking place via initiatives to promote screening, brief intervention and referral to treatment (aka SBIRT). Electronic screening and brief intervention (eSBI) is a subset of SBIRT and essentially takes SBIRT into an electronic medium suitable for use in primary care and other generalist settings. The Community Prevention Services Task Force (CPSTF) recommends eSBI as an evidence-based intervention for the prevention of excessive alcohol consumption [12]. Most studies to date have tested this approach in health care (e.g., emergency room) and university settings with adults and young adults and for relatively short follow-up time (i.e., 6-months post intervention). In these settings, the CPSTF found sufficient evidence of efficacy across studies and age groups [12]. Little trial data exists for the use of eSBI in adolescents, minority racial/ethnic groups, or for longer periods of follow-up. To date no studies have tried to extend this model to populations such as YMSM and YTW, in community-based settings in which these youth tend to access services.

To capitalize on this opportunity, we propose to test a structural change to the HIV prevention and care continuum by integrating substance use screening and brief intervention into the traditional community-based HIV testing environment. We will couple standard HIV prevention with eSBI for at the point of HIV testing.

### Study objectives

The purpose of this study is to assess the feasibility, acceptability, and initial efficacy of eSBI in comparison to an electronic attention control intervention (i.e., promotion of good nutrition), coupled with standard HIV prevention, on alcohol use among YMSM and YTW in community-based HIV testing environments in Chicago. Secondary objectives are to assess intervention effects on sexual behavior, as well as engagement within the HIV prevention and care continuum, and to assess modification of the intervention effect by co-morbid mental health problems (i.e., symptoms of depression, anxiety, and exposure to trauma).

## Methods/design

### Design

**Fig. 1:**
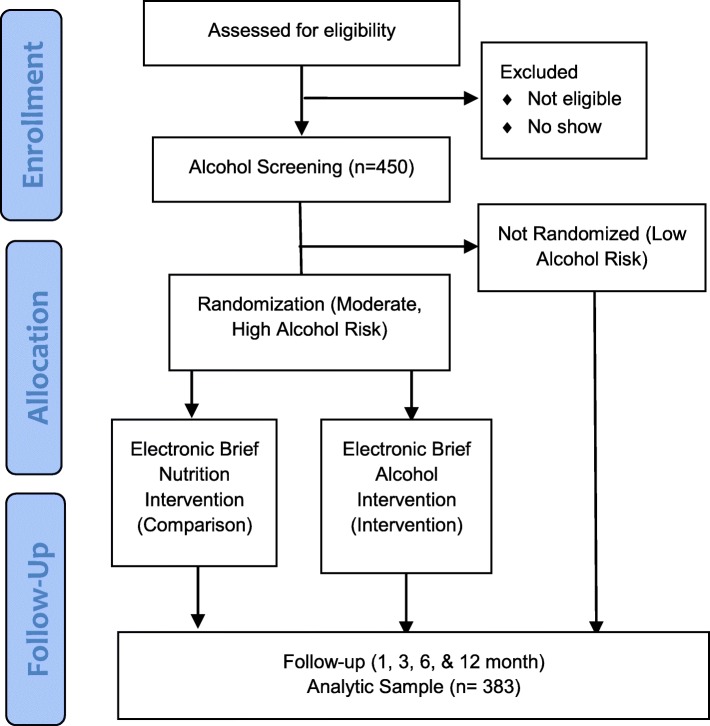
Step up, Test Up CONSORT Diagram

This study is an individually randomized controlled trial (RCT) of eSBI among youth vulnerable to HIV infection. Using an electronic portal, all participants are screened for alcohol misuse and receive immediate feedback regarding their level of use (e.g., how their use compares to others, whether it exceeds “safe use” guidelines); those who screen for moderate to high alcohol use, including binge drinking, are then randomized to either electronic intervention or control modules. All participants, regardless of randomization status, are followed for 12 months with in-person visits conducted at 1, 3, 6 and 12-month intervals (Fig. 1).

### Identification and recruitment of participants

Youth are recruited from HIV testing centers at the three primary sites in Chicago: the Division of Adolescent Medicine at Lurie Children’s Hospital, Howard Brown Health, and the Village at the University of Chicago. Individuals are eligible if they are: (a) aged 16 to 25 years, (b) interested in testing for HIV infection and (c) HIV-negative or HIV status unknown (per self-report; verified at the point of HIV testing), and (d) are MSM or a transgender woman who has sex with men (i.e., born male, identify as female/transgender, and at any point in the gender transition process); (e) English-speaking.

### Randomization

Upon enrollment, all participants complete HIV testing and screen for alcohol misuse using the Alcohol Use Disorders Identification Test (AUDIT) via an electronic portal, then receive immediate feedback regarding their level of use. Participants who screen moderate to high risk for alcohol misuse or endorse binge drinking on the AUDIT are randomized via computerized assignment in the portal (1:1) to either brief intervention modules to reduce alcohol misuse or a time-and attention-matched control (i.e., promotion of good nutrition). Study staff are blinded to random assignments.

### Description of the intervention: eSBI

Those randomized to the intervention complete modules on 11 topical areas (see Table 1), each with a single webpage, in an MI format. The intervention content was targeted to late adolescents and young adults in terms of voice and language (e.g., open, non-authoritarian, inclusive), contexts (e.g., school as well as work contexts) and reading level. Completion of the intervention is expected to take 10–15 min. The electronic intervention and control modules are delivered using a customized eSBI platform developed by Radiant Interactive Group (Laguna Niguel, CA). Radiant uses industry-standard data encryption and security to manage Protected Health Information recorded by their system.

**Table 1:** eSBI Topical Areas

Webpage	Topic
1	Importance of change
2	Downsides of drinking
3	Confidence scale
4	Barriers to change
5	Explore ways to cut back or quit
6	Strategies for cutting back
7	Change motivators
8	1 year from now
9	Readiness to change
10	Summary slide
11	More information and/or referral

### Time- and attention-matched control: diet and nutrition

Those randomized to control complete a brief time-matched attention control intervention, also in MI format, of equal length, which encourages good nutrition.

### Standard-of-care prevention services

The standard HIV preventive care at each site includes youth-specific HIV testing and counseling and linkage navigation to PrEP or HIV care.

### Study assessments

Participants complete a baseline study visit comprised of standardized assessment via computer-assisted self-interviewing (CASI). Follow-up CASI assessments are conducted at 1, 3, 6, and 12-month follow-ups visits. Detailed contact information is collected in order to facilitate study retention.

Primary Outcome: Change in the frequency/quantity of self-reported recent alcohol use at 1, 3, 6 and 12-months on the Daily Drinking Questionnaire [13].

Secondary Outcomes: Sexual risk behavior is measured via a modified version of the AIDS-Risk Behavior Assessment [14] used in prior studies of YMSM and YTW [15, 16]. Engagement in HIV and/or PrEP care is measured via medical records abstraction of clinic visit continuity (i.e., at least two clinic visits over the 12-month observation period).

Moderators: Depression, anxiety, and trauma will be measured using the short version of the Center for Epidemiologic Studies Depression Scale (CES-D10), [17] 7-item Generalized Anxiety Disorder (GAD-7) [18] and Life Events Checklist (LEC), [19] respectively.

### Statistical analysis

Several data quality checks will be conducted prior to analysis, including analysis of descriptive statistics and graphic plots to detect appropriate range of variables and to detect missing data or invalid cases. All data are stored in a password-protected network server with daily back-up. In order to assess the initial efficacy of eSBI, the intervention and control groups will be compared on baseline characteristics to assess randomization, and variables for which there are significant group differences will be controlled in subsequent analyses. Extensions of generalized linear models (GLMs) will be used to assess the effect of the intervention on the primary alcohol-related outcomes at 1, 3, 6 and 12-month follow-up visits (post-intervention). Intervention effects will be examined in terms of average group differences over follow-up and using models with an indicator for intervention group, time, and a group by time interaction to determine whether the intervention and control groups differ in response to the intervention over time. Potential confounding and effect modification by sociodemographic characteristics and co-morbid conditions will be examined in exploratory analysis, and these variables will be selected for inclusion in multivariable models based on statistical significance and conceptual importance. All models will account for correlation among repeated measures on individuals over time as appropriate (e.g., by inclusion of subject-level random effects). Potential modifiers of the intervention effect on outcomes will be examined using models including two-and three-way interactions of intervention group, time, and moderator effects to determine whether intervention effects differ across levels of the modifying variables.

### Sample size calculation

We propose to enroll 450 participants and have estimated 80% power to detect effects for our primary (alcohol use) and secondary (PrEP uptake) outcomes using formulas for longitudinal study designs with attrition [20]. Calculations assume an exchangeable covariance structure, measurements at baseline and 4 follow up points, and within-subject correlation among repeated measurements (rho) of 0.5. All estimates use two-sided tests of significance and alpha of 0.05. With a retention rate of 85% at 1 year, we would have > 80% power to detect effect sizes (Cohen’s d) of 0.23 (small effect size) [21] or greater for between-group comparison of continuous measures of alcohol consumption and other substance use. For binary outcomes (i.e. any binge drinking), we would have ≥80% power to detect differences in proportions of 10% or greater when the proportion in the control group ranges from 25 to 30%, using a repeated measures framework with 4 follow-up measures and assumptions stated above. Thus, this study is powered to detect small to medium effect sizes that are consistent with or smaller than others reported in the literature [22].

## Discussion

We describe herein the design of a randomized controlled trial of an eSBI, “Step Up, Test Up,” to reduce alcohol misuse among youth, ages 16–25 who are vulnerable to HIV infection in Chicago. The intervention draws on MI-informed approaches, including SBIRT, adapted in an electronic medium suitable for use in primary care and other generalist settings. We hope to extend the evidence base for eSBI to a group vulnerable to alcohol misuse, but with limited opportunity for intervention in traditional settings. We believe the design of this study has several strengths, including its focus on a younger group vulnerable to both alcohol misuse and related consequences, using a randomized controlled design in community-based practice environments where youth often seek testing for HIV infection. Furthermore, we believe that this intervention is developmentally responsive and addresses the age range with the steepest rise in both alcohol misuse and HIV acquisition (i.e, ages 16–25 years).

Step Up, Test Up, is among the first interventions to be tested in a rigorous trial of sufficient size to detect effects of eSBI and targeting youth who are vulnerable to alcohol misuse and HIV infection. We consider it a potential innovation in the HIV testing environment, which given the brief and electronic format, is well positioned for scale. It was designed to meet the standard for evidence-based interventions and to extend the base of evidence for intervention with this population with many assets, yet increased susceptibility to HIV and other STIs.

## Data Availability

Not applicable.

## Abbreviations

ARBA: AIDS Risk Behavior Assessment
AUDIT: Alcohol Use Disorders Identification Test
CASI: Computer Assisted Self Interviewing
CDC: Centers for Disease Control and Prevention
CES-D10: Center for Epidemiological Studies Depression 10-item Scale
CPSTF: Community Prevention Services Task Force
eSBI: Electronic Screening and Brief Intervention
GAD-7: Generalized Anxiety Disorder 7-item Scale
GEE: Generalized Estimating Equations
GLM: Generalized Linear Models
HIV: Human Immunodeficiency Virus
LEC: Life Events Checklist
MI: Motivational Interviewing
NIH: National Institutes of Health
PrEP: Pre-Exposure Prophylaxis
RCT: Randomized Controlled Trial
SBI: Screening & Brief Intervention
SBIRT: Screening, Brief Intervention & Referral to Treatment
US: United States
YMSM: Young Men Who Have Sex with Men
YTW: Young Transgender Women
